# Loss of MMP9 leads to altered inflammation and ECM interactions in a mouse model of cerebral small vessel disease

**DOI:** 10.1002/alz70855_105564

**Published:** 2025-12-24

**Authors:** Joshua Lykins, Sherika N. Johnson, Katelynn E. Krick, Tiffany L. Sudduth, Colin B Rogers, Erica M. Weekman, Donna M. Wilcock

**Affiliations:** ^1^ Indiana University School of Medicine/Stark Neurosciences Research Institute, Indianapolis, IN, USA; ^2^ Indiana University School of Medicine, Stark Neurosciences Research Institute, Department of Anatomy, Cell Biology, and Physiology, Indianapolis, IN, USA; ^3^ University of Kentucky / Sanders‐Brown Center on Aging, Lexington, KY, USA; ^4^ University of Kentucky College of Medicine, Sanders‐Brown Center on Aging, Lexington, KY, USA; ^5^ Indiana University School of Medicine, Stark Neurosciences Research Institute, Department of Neurology, Indianapolis, IN, USA

## Abstract

**Background:**

Vascular contributions to cognitive impairment and dementia (VCID) represent a major cause of dementia. Hyperhomocysteinemia (HHcy)‐driven cerebral small vessel disease (cSVD) leads to the degeneration of astrocytic end‐feet and an upregulation of matrix metalloproteinases (MMPs), which remodel the basement membrane, triggering growth factor activation and inflammation. This study explores the role of MMP9 in cSVD using MMP9^‐/‐^ mice subjected to a HHcy diet.

**Method:**

Six‐month‐old C57Bl6/J mice and MMP9^‐/‐^ mice were placed on a diet deficient in B vitamins and enriched in methionine or a control diet with normal levels of B vitamins and methionine for 12 weeks. The left hemibrain was fixed in PFA while the right brain was dissected for biochemistry. RNA was extracted from the frontal cortex and analyzed using NanoString's Mouse Neuroinflammation and Cardiovascular Disease panels. Microhemorrhage assessment via Prussian blue staining as well as Dp71, AQP4, and GFAP detected by immunohistochemistry were performed on the left hemisphere.

**Result:**

Gene expression analysis in MMP9^‐/‐^ mice on a control diet revealed significant reductions in inflammation‐related genes (Cd14, Slamf8, Prkcq, Lag3) and lower expression of the microglial marker P2ry12 compared to wild‐type mice. Additionally, MMP9^‐/‐^ mice exhibited increased expression of macrophage extracellular matrix (ECM) scavenging receptors (Cd44, Cd163, Siglec1) and enhanced regulation of the PIP3 signaling pathway across multiple cell types. Under a HHcy diet, wild‐type mice showed elevated expression of Cpa3, Reln, and the glial transcription factor EOMES, while MMP9^‐/‐^ mice demonstrated increased expression of PECAM1, Hira, Myd88, and Pink1. Although MMP9^‐/‐^ mice displayed a trend toward fewer microbleeds, the difference was not statistically significant.

**Conclusion:**

Our findings suggest that MMP9 plays a key role in the inflammatory response associated with HHcy‐induced cSVD. The absence of MMP9 led to reduced expression of several inflammation‐related genes as well as an increase in ECM interacting genes from macrophages, though differences in gene expression profiles between wild‐type and MMP9^‐/‐^ mice highlight distinct molecular pathways. Further studies are needed to fully understand MMP9's role in cSVD progression.

